# Revealing the novel ferroptosis-related therapeutic targets for diabetic foot ulcer based on the machine learning

**DOI:** 10.3389/fgene.2022.944425

**Published:** 2022-09-26

**Authors:** Xingkai Wang, Guidong Jiang, Junwei Zong, Decheng Lv, Ming Lu, Xueling Qu, Shouyu Wang

**Affiliations:** ^1^ Department of Orthopedic Surgery, The First Affiliated Hospital of Dalian Medical University, Dalian, China; ^2^ Dalian Medical University, Dalian, China; ^3^ College of Integrative Medicine, Dalian Medical University, Dalian, China; ^4^ Trauma and Tissue Repair Surgery Department, Dalian Municipal Central Hospital, Dalian, China; ^5^ Pelvic Floor Repair Center, The Affiliated Dalian Maternity Hospital of Dalian Medical University, Dalian, China; ^6^ Pelvic floor repair center, Dalian Women and Children Medical Center (Group), Dalian, China

**Keywords:** DFU, WGCNA, ferroptosis, machine learning, prediction model

## Abstract

**Objectives:** DFU is a serious chronic disease with high disability and fatality rates, yet there is no completely effective therapy. While ferroptosis is integrated to inflammation and infection, its involvement in DFU is still unclear. The study aimed to identify ferroptosis-related genes in DFU, providing potential therapeutic targets.

**Methods:** In the GEO database, two DFU microarray datasets (GSE147890 and GSE80178) were collected. WGCNA was conducted to identify the modular genes most involved in DFU. Subsequently, enrichment analysis and PPI analysis were performed. To yield the DFU-associated ferroposis genes, the ferroposis genes were retrieved from the FerrDb database and overlapped with the modular genes. Eventually, an optimal DFU prediction model was created by combining multiple machine learning algorithms (LASSO, SVM-RFE, Boruta, and XGBoost) to detect ferroposis genes most closely associated with DFU. The accuracy of the model was verified by utilizing external datasets (GSE7014) based on ROC curves.

**Results:** WGCNA yielded seven modules in all, and 1223 DFU-related modular genes were identified. GO analysis revealed that inflammatory response, decidualization, and protein binding were the most highly enriched terms. These module genes were also enriched in the *ErbB* signaling, *IL-17* signaling, *MAPK* signaling, growth hormone synthesis, secretion and action, and tight junction KEGG pathways. Twenty-five DFU-associated ferroposis genes were obtained by cross-linking with modular genes, which could distinguish DFU patients from controls. Ultimately, the prediction model based on machine learning algorithms was well established, with high AUC values (0.79 of LASSO, 0.80 of SVM, 0.75 of Boruta, 0.70 of XGBoost). *MAFG* and *MAPK3* were identified by the prediction model as the most highly associated ferroposis-genes in DFU. Furthermore, the external dataset (GSE29221) validation revealed that *MAPK3* (AUC = 0.81) had superior AUC values than *MAFG* (AUC = 0.62).

**Conclusion:** As the most related ferroptosis-genes with DFU, *MAFG* and *MAPK3* may be employed as potential therapeutic targets for DFU patients. Moreover, *MAPK3*, with higher accuracy, could be the more potential ferroptosis-related biomarker for further experimental validation.

## Introduction

Diabetic foot ulcer (DFU), one of the most devastating consequences of diabetes, is a major health concern that places a serious financial, physical, and mental burden on sufferers. Extensive diabetic alterations, such as neuropathy and vascular disease, frequently aggravate the course of DFU (Teena et al., 2020). Even with recent advances in DFU treatment, a considerable proportion of individuals experience chronic trauma as a result of an irreversible process (Chen et al., 2016). Thus, it is critical to investigate the molecular mechanisms of DFU and inhibit the creation of chronic trauma in order to improve the treatment efficiency and prognosis of DFU patients.

Ferroptosis is an iron-dependent programmed cell death that differs from apoptosis, pyroptosis, and necrosis, being characterized by excessive iron accumulation and elevated lipid peroxidation (Stockwell et al., 2017). Induction of ferroptosis in cancer cells has been proven to be a viable alternative treatment for tumor disorders that are resistant to conventional treatments (Hassannia et al., 2019). Since ferroptosis is reported to be correlated with a variety of disorders, it has the potential to play a role in the etiology of DFU. However, the particular regulation mechanism of ferroptosis in DFU is unknown and requires further investigation.

In the current study, we employed WGCNA to identify potential genes in ferroptosis genes linked to specific genes closely associated to DFU. Finally, utilizing multiple machine learning methods, a robust prediction model for identifying DFU patients was established and validated by employing an external DFU dataset. We investigated the genetic connection among ferroptosis with DFU. The DFU-related ferroptosis genes could be employed as biomarkers for disease diagnosis and therapy monitoring, as well as a reference for early therapeutic targets for DFU.

## Materials and methods

### Microarray data download and data preprocessing

**FIGURE 1:**
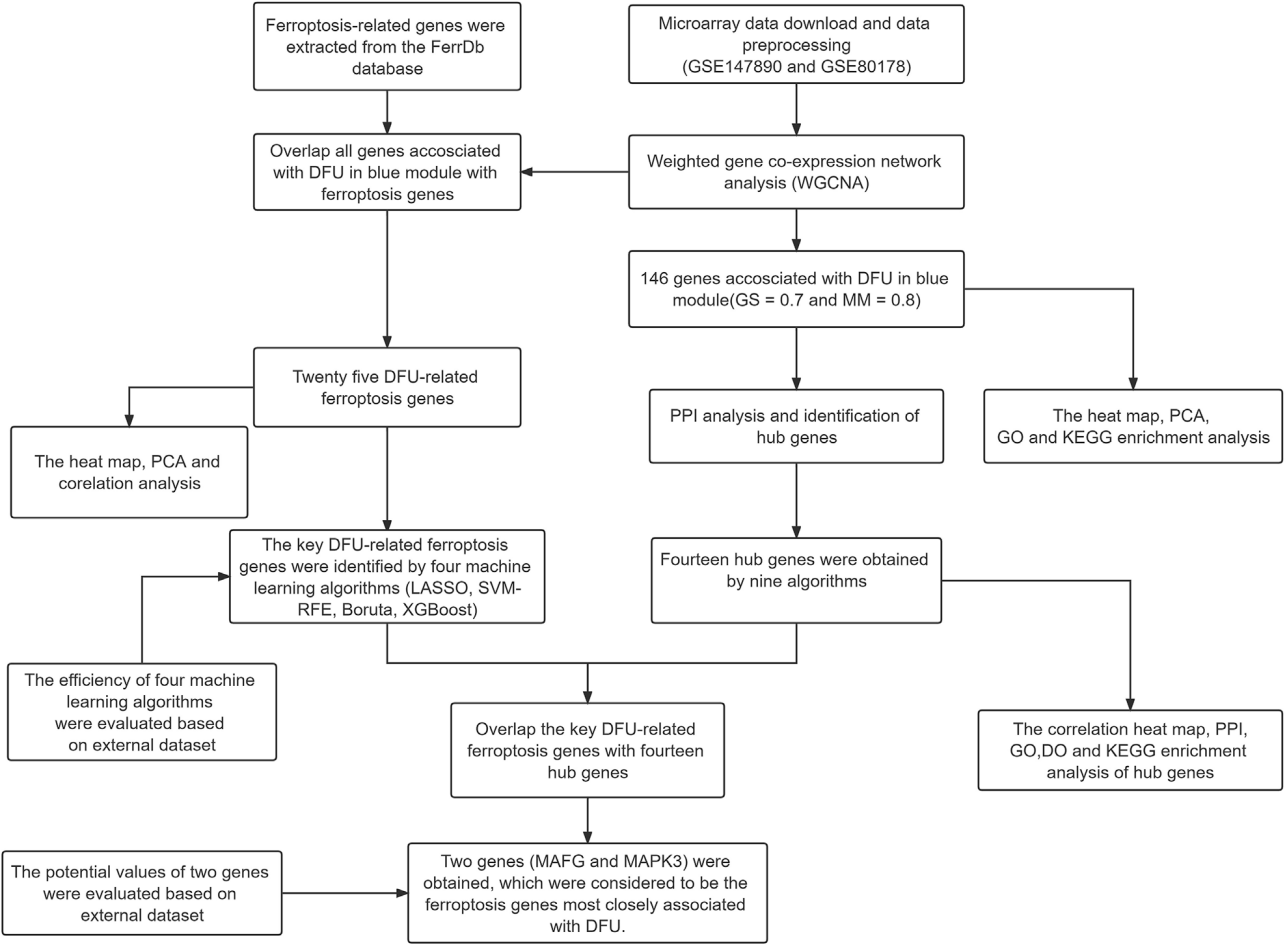
The workflow chart of data preparation, processing, analysis, and validation.

We used the “*GEOquery*” package of R software (version 4.1.2, http://r-project.org/) to download the DFU sample source from the Gene Expression Omnibus (GEO) (https://www.ncbi.nlm.nih.gov/geo/) database. The DFU-related expression profiles GSE147890, GSE80178, GSE7014, and GSE29221 were all from Homo sapiens (Table 1). The GSE147890 and GSE80178 gene expression matrices were then combined to remove inter-batch differences using the “sva” package and to drop samples where inter-group differences could not be removed between groups (Deng et al., 2020). A flow diagram of the study is shown in Figure 1.

**TABLE 1:** Details for datasets.

Data set	Category	Annotated from	Count	Annotations
Driver	Regulator	Gene	108	150
Suppressor	Regulator	Gene	69	109
Marker	Marker	Gene	111	123
Inducer	Regulator	Small molecule	35	54
Inhibitor	Regulator	Small molecule	41	46
Ferroptosis aggravates disease	Ferroptosis-disease association	Ferroptosis and disease	49	58
Ferroptosis alleviates disease	Ferroptosis-disease association	Ferroptosis and disease	46	77

### Weighted gene co-expression network analysis.

Firstly, a soft threshold for network construction was selected to construct a gene co-expression network by an R package called “weighted gene co-expression network analysis (WGCNA)” (Wang et al., 2022). The adjacency matrix was constructed by weighting coefficients, and the adjacency matrix was a continuous value between 0 and 1, which conformed to the power-law distribution and was closer to the real biological state. Secondly, a scale-free network was constructed to hierarchically cluster the modules, identify the gene co-expression modules, and assign them to different colors for visualization. Finally, the correlation between sample phenotypes and each module was assessed by Pearson correlation analysis, and the modules with the closest DFU were screened out. The genes most closely associated with DFU were selected according to the appropriate Gene Significance (GS) for DFU and Module Membership (MM) in this module.

### DFU-related gene analysis and enrichment analysis.

The “ComplexHeatmap” package was used to heat map DFU-related genes and assess the efficiency of genes by principal component analysis (PCA). The Gene Ontology (GO) analysis, including biological processes (BP), molecular functions (MF), and cellular components (CC), was a widely used functional enrichment method. Kyoto Encyclopedia of Genes and Genomes (KEGG) was a database that stores a large number of biological functions, genomes, chemicals, and drug-related pathways. The Database for Annotation, Visualization and Integrated Discovery (DAVID, https://david.ncifcrf.gov/) (Huang et al., 2009a; Huang et al., 2009b) database was used for GO and KEGG enrichment analysis of DFU-related genes (Zheng et al., 2020), and the results were visualized using the “ggplot2” package.

### Protein-protein interaction creation and identification of hub genes.

Search Tools for the Retrieval of Interacting Genes (STRING, http://string-db.org) (Szklarczyk et al., 2021) was an online tool for predicting the protein-protein interaction (PPI), which was implemented to construct a PPI network with a confidence score of >0.40 and visualize the network model with Cytoscape V3.8.0 software. Nine algorithms (Betweenness, Radiality, MNC, EPC, DMNC, MCC, Degree, Clustering Coefficient, Closeness) in the CytoHubba plugin were used to evaluate the importance of each node and to select the common genes in the top 30 nodes of each algorithm as hub genes (Liu et al., 2020). Subsequently, enrichment analysis of the hub gene including GO, KEGG, and Disease Ontology (DO) was performed by the R package “clusterProfiler” and FDR < 0.05 was considered significant.

### Identification and analysis of ferroptosis-related genes.

Ferroptosis-related genes were obtained for further analysis according to the FerrDb database (http://www.zhounan.org/ferrdb/) (Zhou and Bao, 2020) (Table 2), an artificial database collection of ferroptosis-related markers and diseases. Genes in the intersection of ferroptosis-related genes and those in the modules most closely associated with DFU for WGCNA were taken using the online tool Bioinformatics & Evolutionary Genomics (http://bioinformatics.psb.ugent.be/webtools/Venn/), and genes in the intersection were considered to be specifically expressed in DFU patients with ferroptosis-related genes. Subsequently, correlations between ferroptosis-related genes were assessed using the “corrplot” package. Heat maps and PCA were visualized using R software, respectively.

**TABLE 2:** Details for Ferroptosis genes.

Dataset	Platform	Count	Diabetic	Control
GSE147890	GPL571 [HG-U133A_2] Affymetrix Human Genome U133A 2.0 Array	26	12	14
GSE80178	GPL16686 [HuGene-2_0-st] Affymetrix Human Gene 2.0 ST Array	12	9	3
GSE7014	GPL570 [HG-U133_Plus_2] Affymetrix Human Genome U133 Plus 2.0 Array	36	30	6
GSE29221	GPL6947 Illumina HumanHT-12 V3.0 expression beadchip	24	12	12

### Robust predictive model built using multiple machine learning methods

The R packages “glmnet,” “caret,” “Boruta” and “XGBoost” were used to build a machine learning model (Huang et al., 2022). The least absolute shrinkage and selection operator (LASSO), Support Vector Machine Recursive Feature Elimination (SVM-RFE), Boruta, and extreme gradient boosting (XGBoost) analyses were performed on the entire dataset to screen for key ferroptosis-related genes. In addition, the GSE7014 dataset was used as an external dataset to validate the prediction model, and the prediction efficiency of the model was evaluated by receiver operating characteristic (ROC) curves. Consequently, the intersection genes between genes obtained by model analysis and hub genes were considered the key ferroptosis-related genes associated with DFU. Finally, the “pROC” package was employed to assess the efficiency of key ferroptosis-related genes as therapeutic markers in the GSE29221 external dataset.

## Results

### Data preprocessing

**FIGURE 2:**
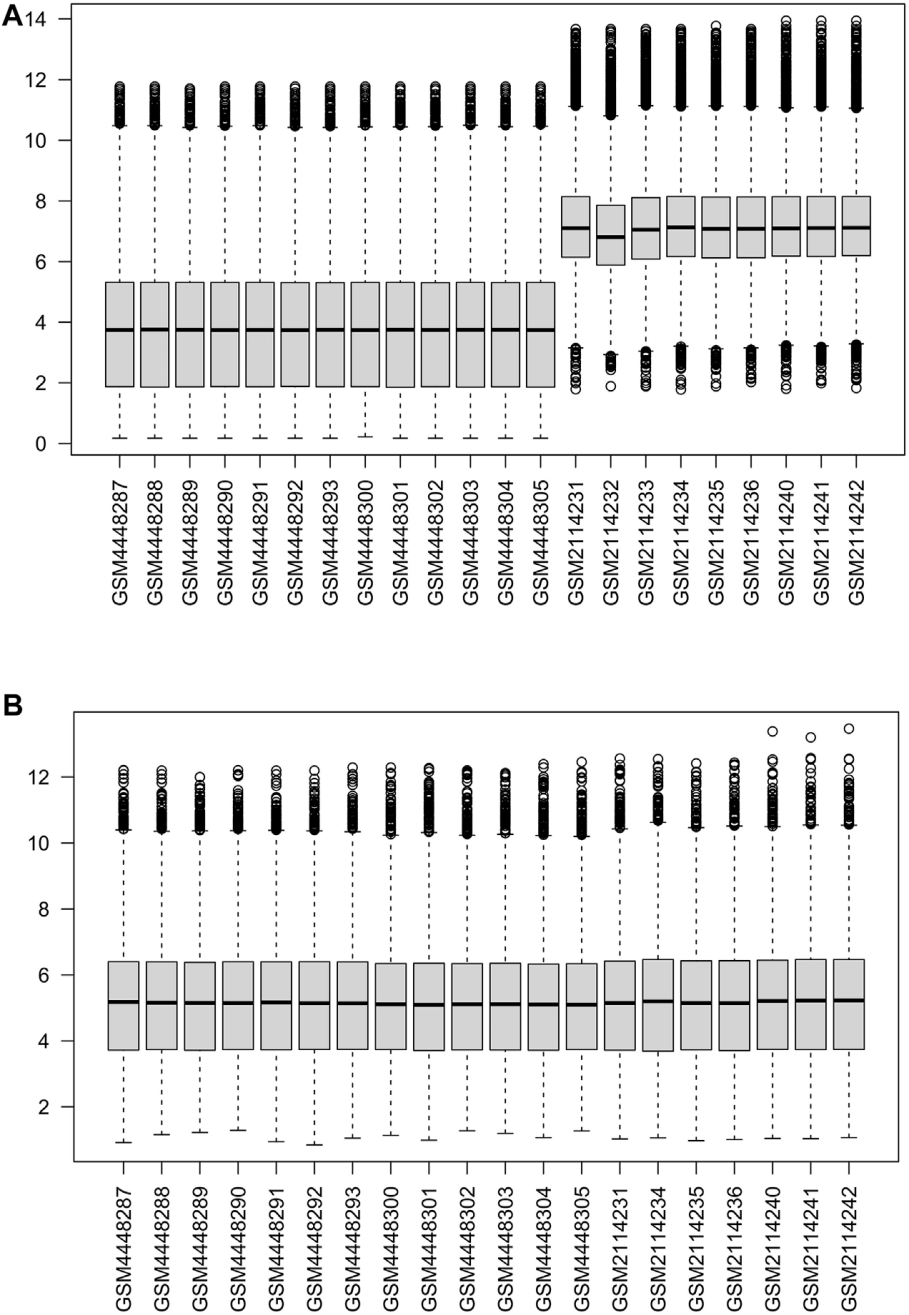
Normalization of gene expression data in samples **(A)**. Before normalization **(B)**. After normalization.

The two data sets GSE147890 and GSE80178 were combined and normalized to remove the batch differences and remove the abnormal samples GSM2114232, GSM2114233 (Figure 2). In addition, the two external datasets GSE7014 and GSE29221 were normalized with an external validation dataset using the “limma” package (Supplementary Figure S1).

### Weighted gene co-expression network analysis and identification of core modules.

**FIGURE 3:**
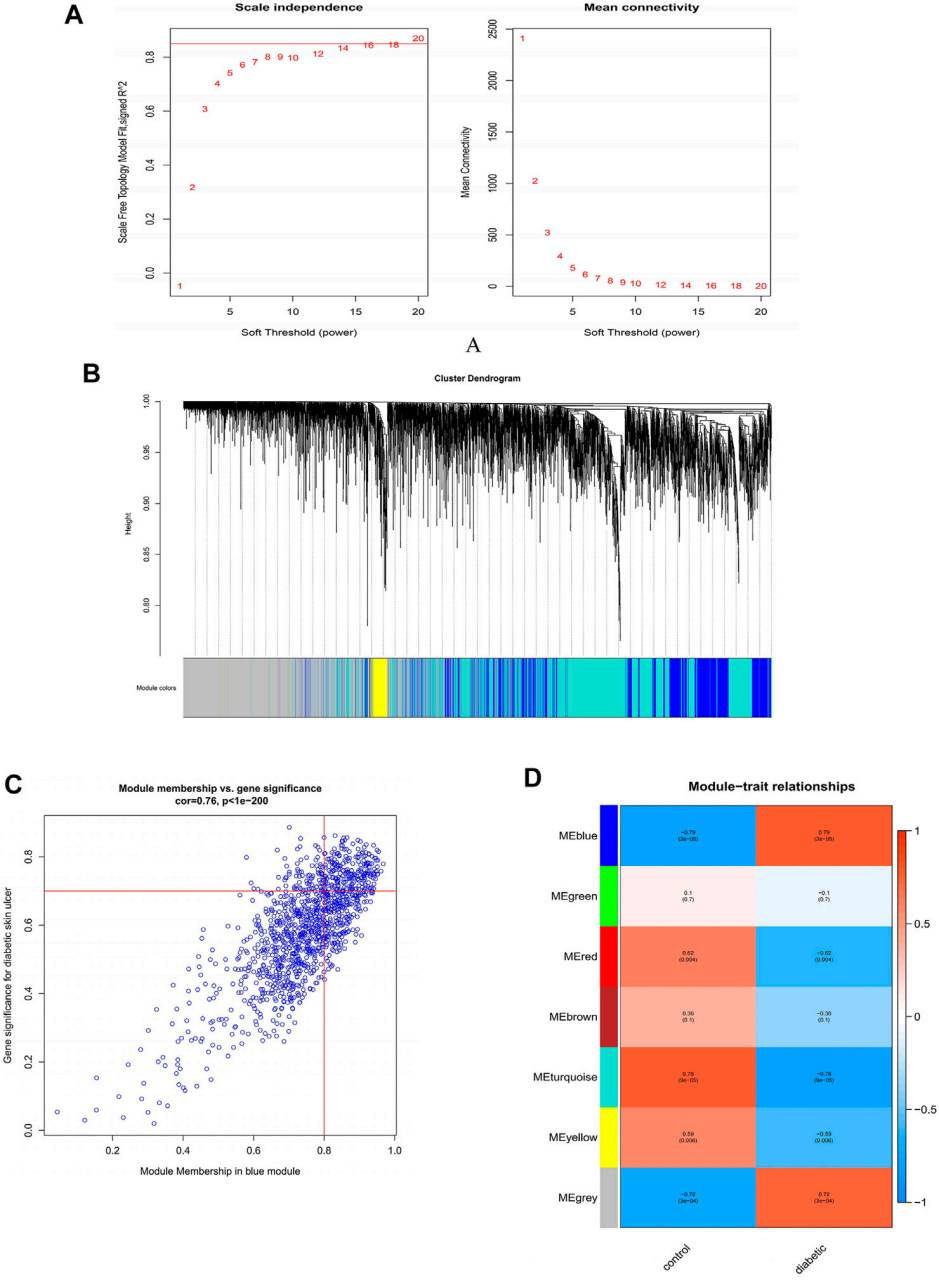
Construction and module analysis of weighted gene co-expression network analysis (WGCNA). **(A)** Network topology analysis under various soft-threshold powers. Left: Analysis of the scale-free index for various soft-threshold powers (β). Right: Analysis of the mean connectivity for various soft-threshold powers. **(B)** Identification of co-expression gene modules. The branches of the dendrogram cluster into seven modules and each one was labeled in a unique color. **(C)** A heatmap showing the correlation between each module eigengene and phenotype. **(D)** The relevance of members in the blue module and DFU.

In order to construct the scale-free network, we chose 20 as a soft threshold (R2 = 0.85) (Figure 3A). Next, the adjacency matrix and the topological overlap matrix were constructed according to the expression matrix. Based on the correlation clustering, the module signature genes that can represent the overall gene expression level of each module were then calculated. A total of seven signature modules were identified and labeled with different colors (Figure 3B). Subsequently, we analyzed the correlation between the modules and the sample phenotypes, and we found the largest correlation among the blue module and DFU (r = 0.79, P = 3e-05) (Figure 3C). The correlation between genes in the blue module and DFU genes was cor = 0.76, p < 1e-200; 146 genes most associated with DFU were selected from this module based on GS = 0.7 and MM = 0.8 (Figure 3D).

### DFU-related gene analysis and enrichment analysis

**FIGURE 4:**
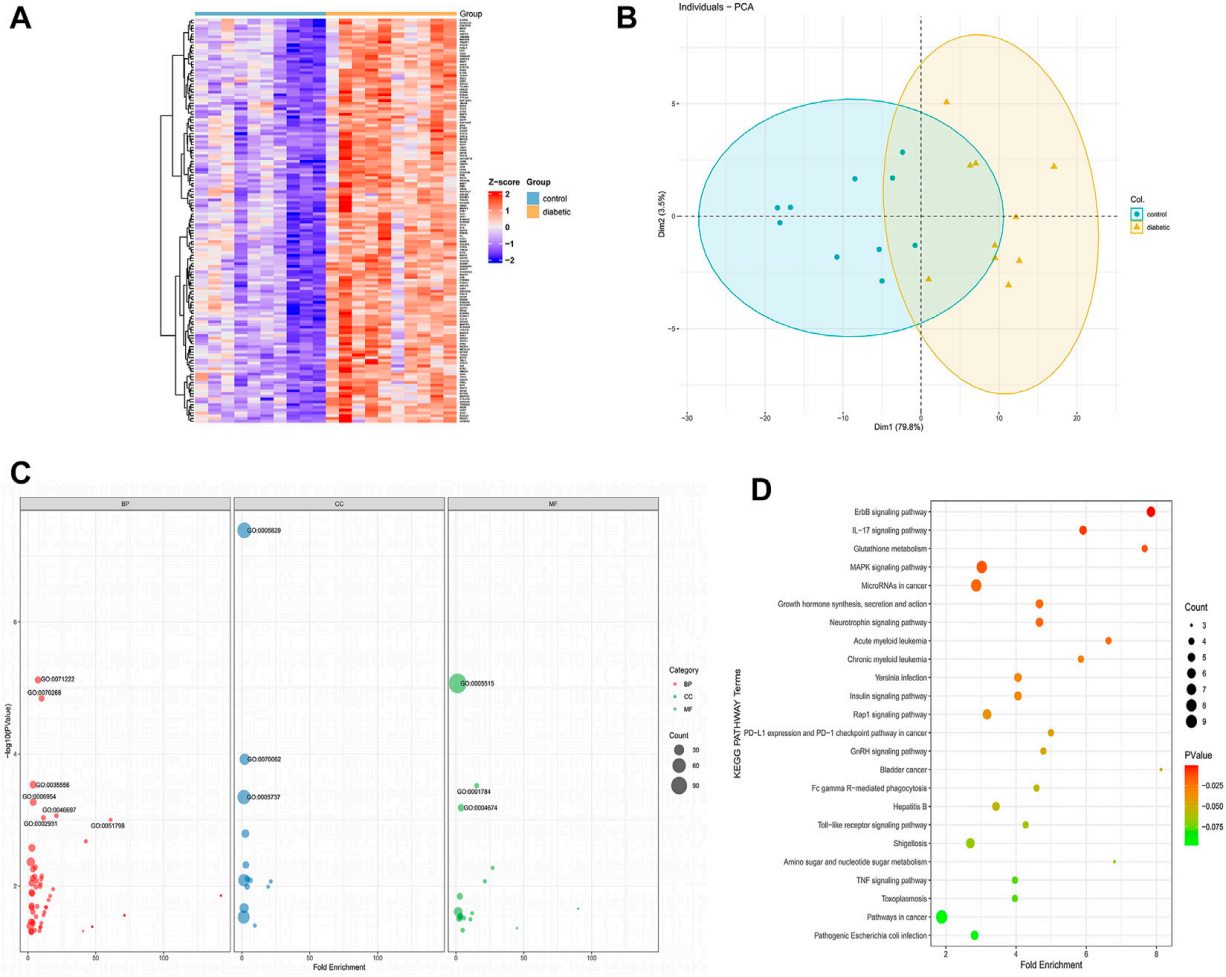
DFU-related genes analysis **(A)**. The heat map of the DFU-related gene expression between DFU samples and control samples (**B)** PCA of DFU-related genes. Principal component 1 (PC1) and principal component 2 (PC2) are used as the X-axis and Y-axis, respectively, to draw the scatter diagram, where each point represents a sample **(C)**. GO analysis divided DFU-related genes into three groups as follows: biological processes (red), cell components (blue), and molecular functions (green). The size of the dot represents the number of gene counts **(D)** KEGG pathway enrichment analysis of DFU-related genes. The size of the dot represents the number of gene counts, and the color of the dot represents the *P*-value.

The 146 DFU-related genes were presented in a heat map to reveal that they were significantly associated with DFU compared to control samples (Figure 4A). PCA indicated that these genes allowed us to distinguish DFU samples from control samples (Figure 4B). The results of the significant GO enrichment analysis of 146 DFU-related genes, including BP, CC, MF, were illustrated in Figure 4C. The results of GO analysis were mainly related to inflammation, protein binding, and kinase activity, such as inflammatory response, coronification, decidualization, protein binding, protein serine/threonine kinase activity, cytosol (Supplementary Table S1). KEGG enrichment analysis demonstrated that these genes were mainly enriched in the *ErbB* signaling pathway, *IL-17* signaling pathway, *MAPK* signaling pathway, growth hormone synthesis, secretion and action, and other pathways related to proliferation and differentiation (Figure 4D). Detailed results of the KEGG analysis are shown in Supplementary Table S2. The results of the enrichment analysis of DFU-related genes suggested that these genes were of interest for our study and could be further investigated.

### Protein-protein interaction analysis and identification of hub genes

**FIGURE 5:**
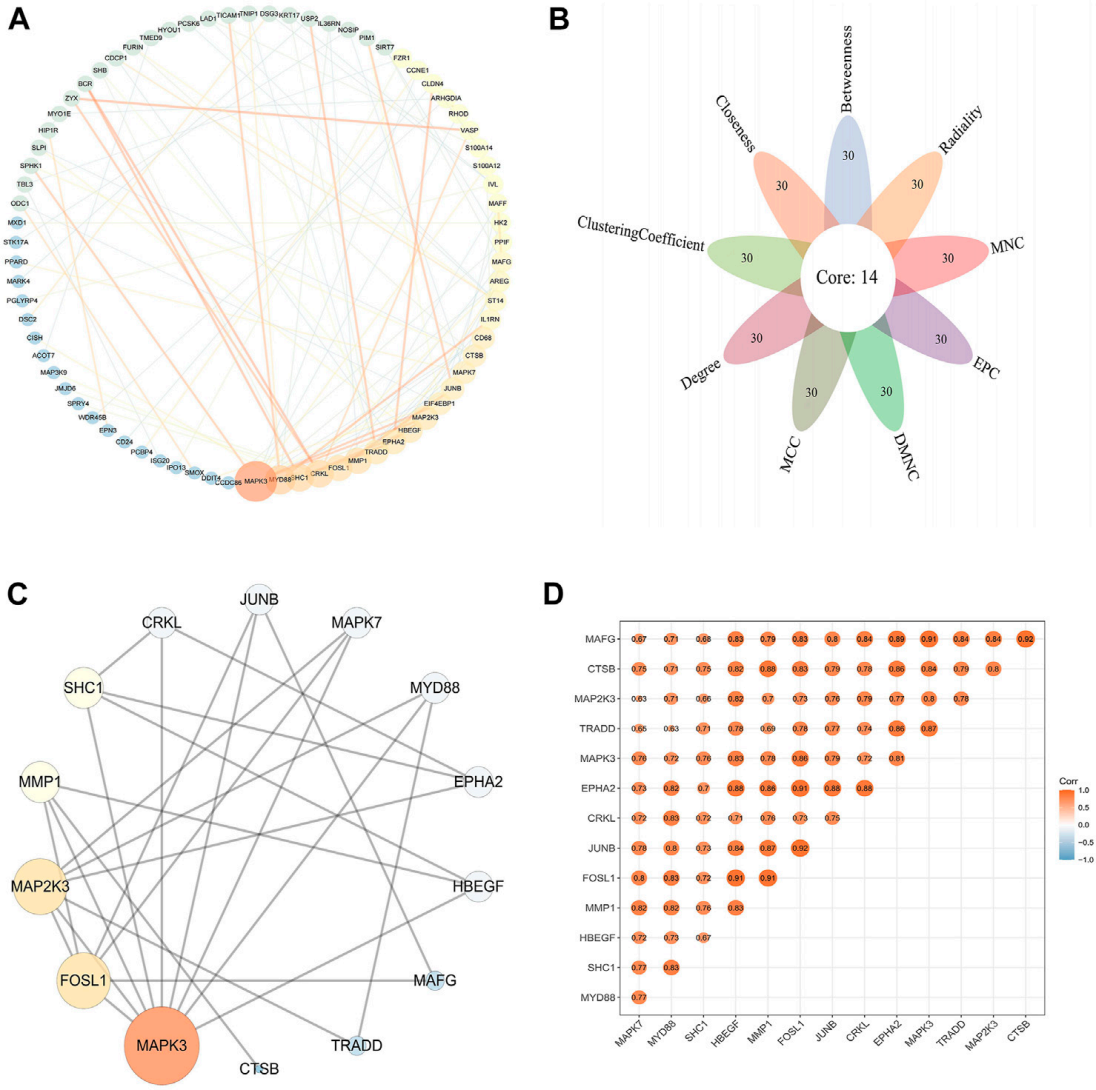
PPI network and hub genes in DFU **(A)** PPI network of DFU-related genes. The size and color of the nodes and edges corresponding to each gene were determined according to the degree and combine-score of interaction, respectively. Color gradients represent the variation of the degrees and combine-score of each gene from high to low **(B)**. Identification of the hub gene. The hub genes were identified by nine algorithms based on CytoHubba **(C)**. PPI network of hub genes. The size and color of the nodes corresponding to each gene were determined according to the degree of interaction. Color gradients represent the variation of the degrees of each gene from high to low **(D)**. Correlation heat map of hub genes. The darker the color, the stronger the correlation.

The PPI data of 146 DFU-related genes were obtained from the STRING database, then the results were visualized and presented using Cytoscape V3.8.0 (Figure 5A). A total of 14 genes were identified as hub genes based on nine algorithms (Betweenness, Radiality, MNC, EPC, DMNC, MCC, Degree, Clustering Coefficient, Closeness), which were common to the top 30 genes in these algorithms (Figure 5B). The PPI network of 14 hub genes was presented in Figure 5C. Subsequently, we used the “ggcorrplot” package to plot the correlation heat map between hub genes, and the results indicated that the genes were positively correlated with each other (Figure 5D).

### Enrichment analysis of hub genes

**FIGURE 6:**
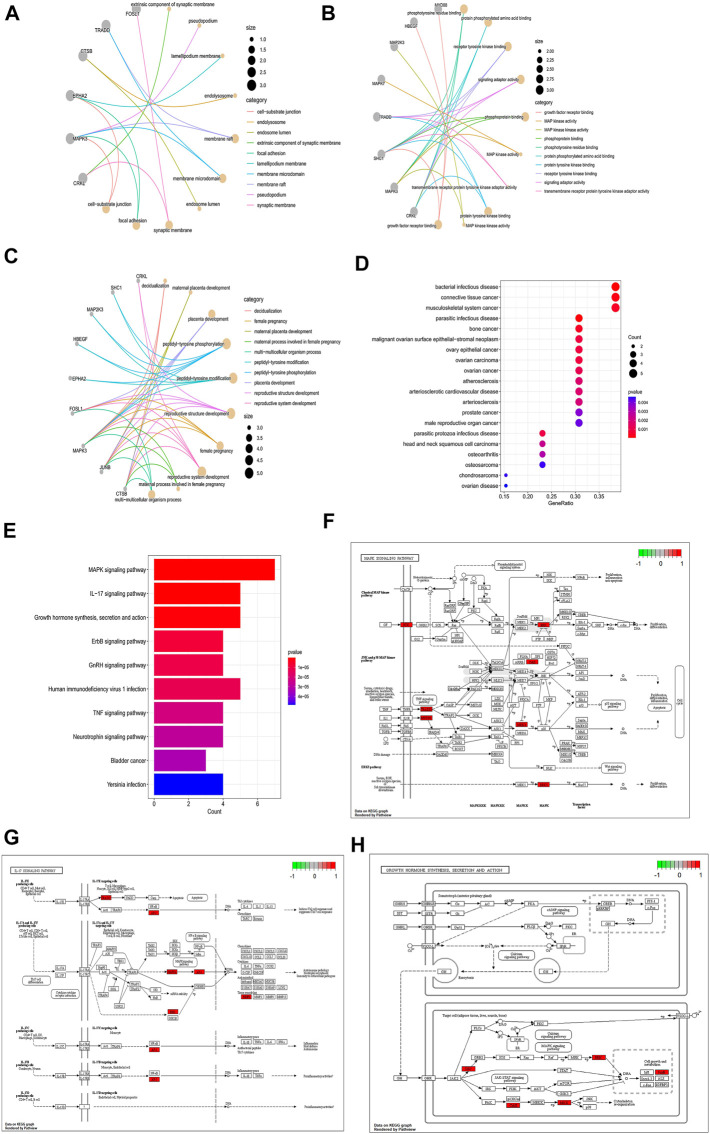
Enrichment analysis of hub genes. **(A–C)** GO enrichment analysis of hub genes (A: BP, **(B)** CC, **(C)** MF). The size of the node respondents the number of the gene counts. **(D)** DO enrichment analysis of hub genes; the size of the dot represents the number of gene counts, and the color of the dot represents the P-value. **(E)** KEGG enrichment analysis of hub genes; the color of the bar res represents the *P*-value.**(F–H)**. *MAPK* signaling pathway; *IL-17* signaling pathway; *MAPK* signaling pathway; Growth hormone synthesis, secretion and action; Red indicates high expression in the pathway, and green indicates low expression in the pathway.

To further analyze the association between hub genes and DFU, the GO, KEGG, and DO enrichment analysis were performed on the hub genes. The results of GO analysis showed that hub genes were mainly related to decidualization, peptidyl-tyrosine phosphorylation, and extrinsic components of the synaptic membrane. Phosphorylated amino acid binding (Figures 6A–C). The DO analysis results were illustrated in Figure 6D. The hub genes were enriched for diseases including bacterial infectious disease, parasitic infectious disease, musculoskeletal system cancer, and other endothelial, infection-related diseases. Moreover, we also submitted the hub genes to KEGG pathway enrichment analysis. As shown in Figures 6E–H, the involved pathways were mainly enriched in the *MAPK* signaling pathway, the *IL-17* signaling pathway, and growth hormone synthesis, secretion and action. The detailed analysis results of GO, DO, and KEGG are shown in Supplementary Table S3.

### DFU-related module genes with ferroptosis-related genes

**FIGURE 7:**
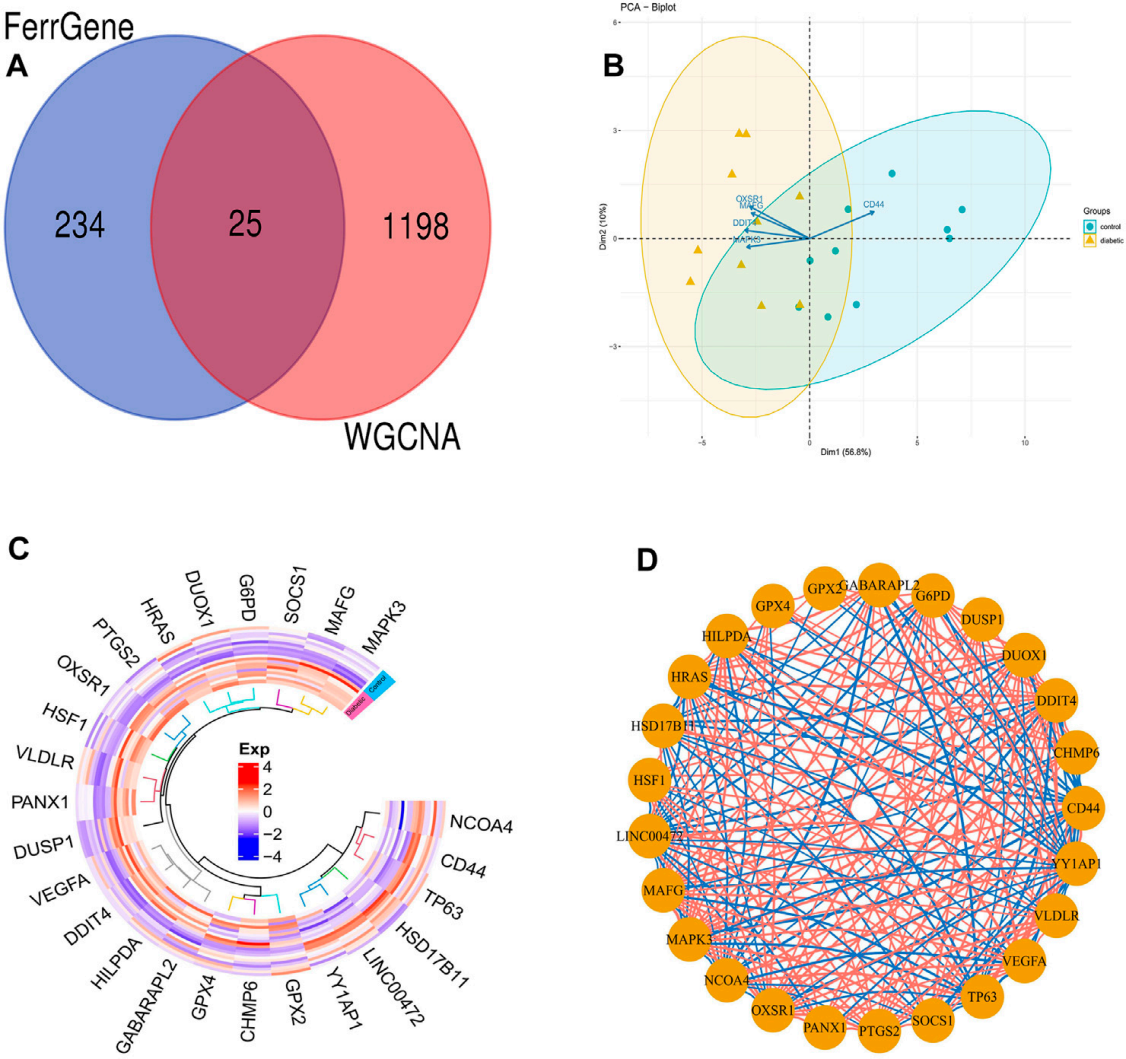
DFU-related ferroptosis genes **(A)**. Venn diagram showing the numbers of overlapped genes between DFU-related module genes and ferroptosis-related genes **(B)**. PCA of DFU-related ferroptosis genes showing good differentiation power **(C)**. The heat map of the DFU-related ferroptosis genes expression between DFU samples and control samples **(D)**. The correlation network of DFU-related ferroptosis genes. The darker the color of the edge, the stronger the correlation.

We overlapped DFU-related genes extracted from the blue module of WGCNA analysis with ferroptosis-related genes extracted from the FerrDb database, and 25 overlapping genes were obtained, namely DFU-related ferroptosis genes, as presented by the Venn diagram (Figure 7A). In addition, PCA revealed that these genes enabled us to effectively distinguish DFU samples from control samples (Figure 7B). Moreover, the heat map drawn could clearly visualize the differences in the expression of these genes among the different samples (Figure 7C). Finally, the correlation network diagram revealed significant inter-correlation between the 25 genes (Figure 7D).

### Construction and validation of an optimal ferroptosis-related dfu prediction model

**FIGURE 8:**
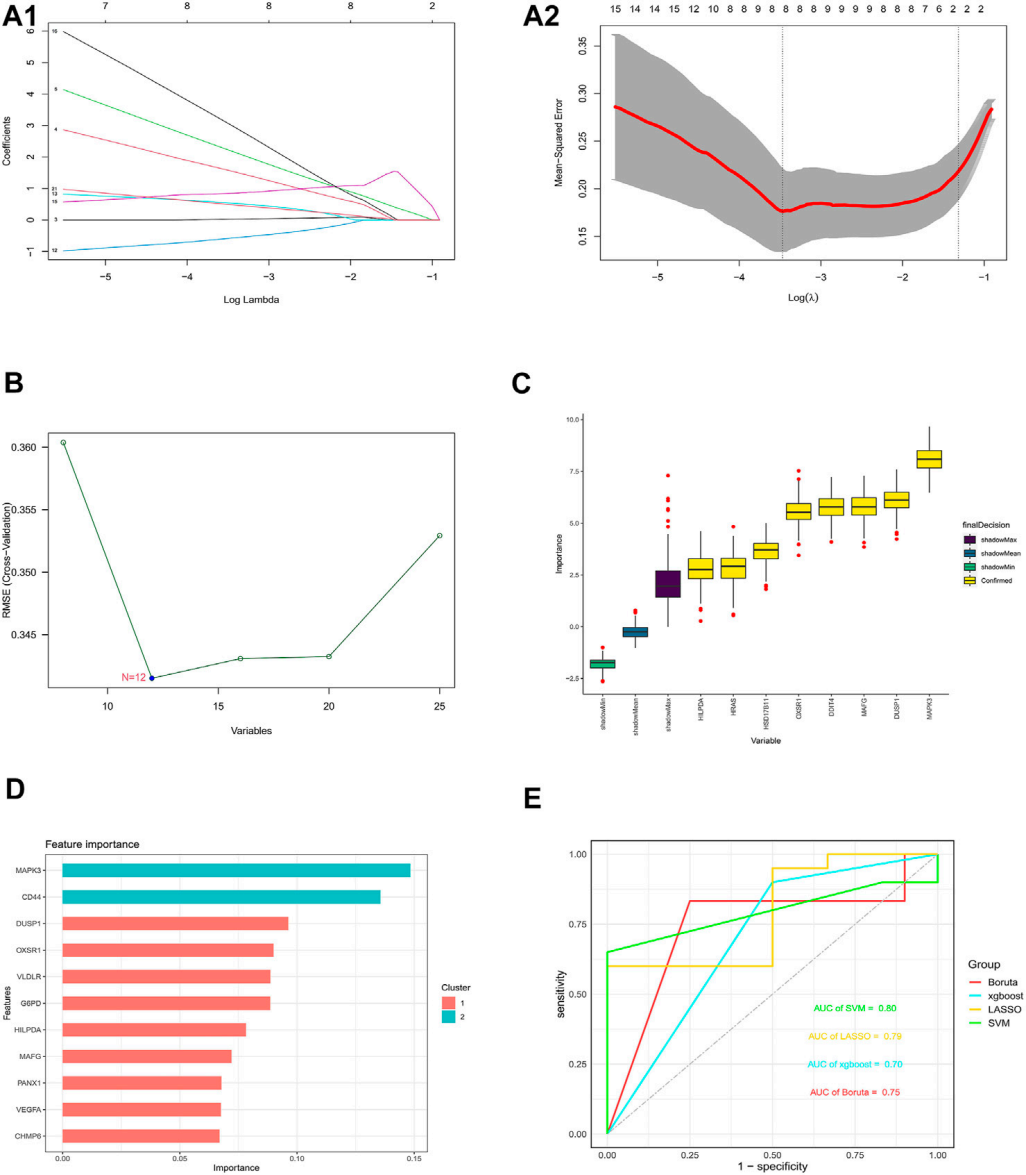
Construction and validation of an optimal ferroptosis-related DFU prediction model **(A)** 8 DFU-related ferroptosis genes obtained using the LASSO algorithm based on the minimum lambda. **(B)** 12 DFU-related ferroptosis genes obtained using the SVM algorithm. **(C)** 8 DFU-related ferroptosis genes obtained using the Boruta algorithm. **(D)** 11 DFU-related ferroptosis genes obtained using the XGBoost algorithm **(E)**. Applying external dataset to validate four predictive models.

Four validated machine learning algorithms (LASSO, SVM-RFE, Boruta, XGBoost) were applied to identify key genes from 25 ferroptosis genes associated with DFU, yielding 8,12,8,11 genes, respectively (Figures 8A–D). Subsequently, we evaluated the efficiency of the four supervised machine learning algorithms using ROC curves based on the external dataset GSE7014, and the AUC values of all four algorithms were greater than 0.7, considering the prediction model results credible (Figure 8E).

### Key ferroptosis genes associated with DFU

**FIGURE 9:**
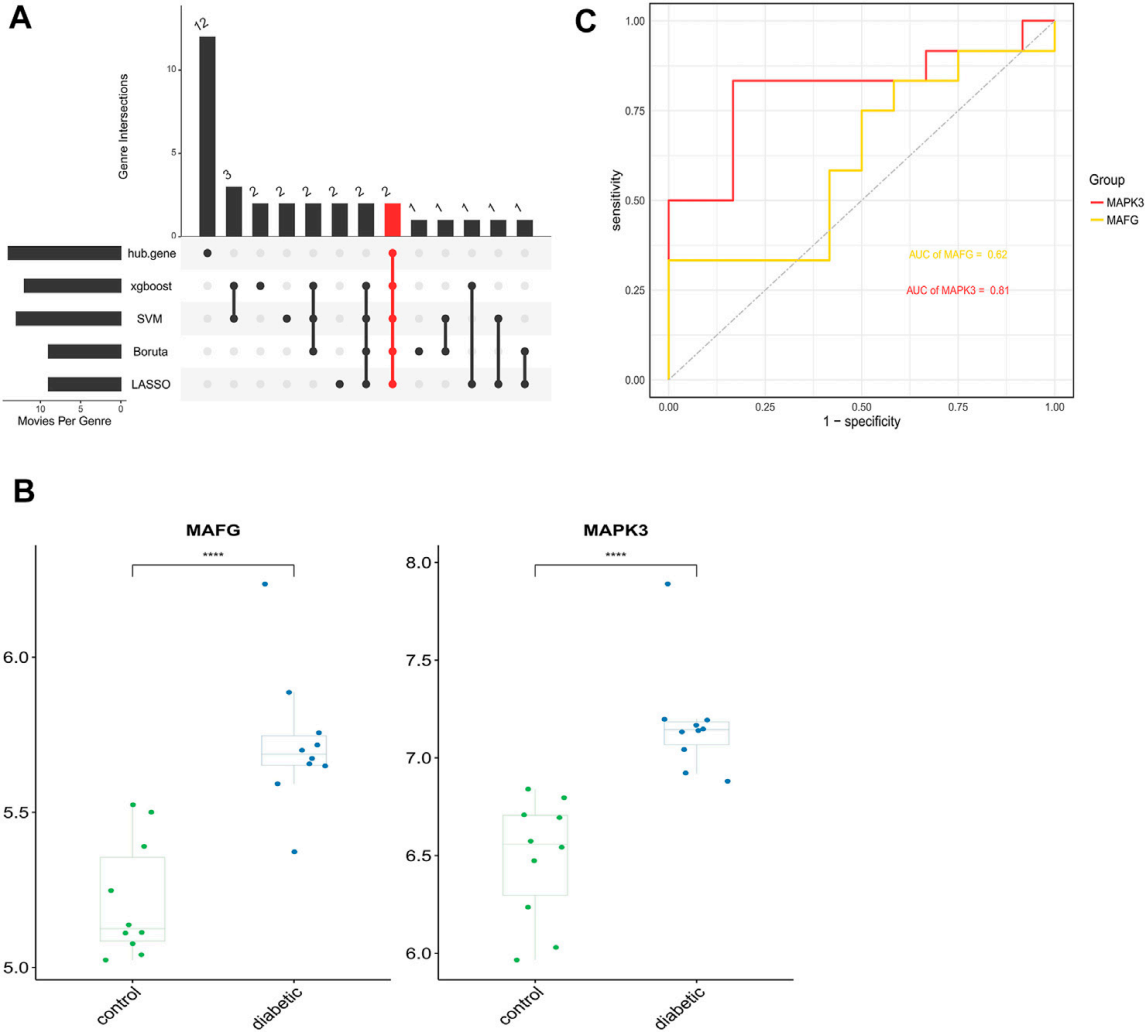
Identification and analysis of key DFU-Related ferroptosis genes **(A)**. Two key DFU-related ferroptosis genes were identified by four machine learning algorithms and f hub genes **(B)**. Expression of the 2 key DFU-related ferroptosis genes in DFU samples and control samples; the differences were statistically significant (****: *p* < 0.0001) **(C)**. Applying external dataset to validate 2 key DFU-related ferroptosis genes.

Genes that commonly belonged to the key ferroptosis genes identified by the four machine learning algorithms and 14 hub genes were considered to be the ferroptosis genes most closely associated with DFU, resulting in two genes (*MAFG* and *MAPK3*) (Figure 9A). Furthermore, the box plot results demonstrated that *MAPK3* and *MAFG* were both highly expressed in DFU patients and low in controls (Figure 9B). Finally, ROC curves were plotted based on the external validation set data to verify the potential of these two genes as therapeutic targets for DFU patients. As shown in Figure 9C, the AUC values of both *MAPK3* and *MAFG* exceeded 0.6, with *MAPK3* (AUC >0.8) being more effective than *MAFG* as a DFU therapeutic target.

## Discussion

DFU is the leading cause of death in the diabetic population, so an urgent need exists to find molecular therapeutic targets with specificity that can help improve the prognosis of patients and reduce mortality. This study applied WGCNA to identify the most relevant model for DFU. Further follow-up analysis was performed on 146 genes in the model based on the set thresholds. Functional annotation identified the primary involvement of these genes in cell proliferation, differentiation, and multiple classical signaling regulatory pathways, including cornification, protein binding, growth hormone synthesis, secretion, and action, and the *MAPK* signaling pathway, indicating that the main biological processes involved in the progression of DFU were cell proliferation and differentiation. In addition, we extracted 14 potential hub genes that contribute most to the diagnosis of DFU. Further enrichment analysis revealed that potential hub genes were also mainly enriched in pathways related to cell proliferation and differentiation.

Ferroptosis is a form of iron-dependent cell death driven by intracellular iron overload and lipid peroxidation (Kuang et al., 2020), and its role in physiological and pathogenic processes has been extensively studied. It has been reported that high concentrations of serum iron may be a risk factor for the development of type 2 diabetes, yet the exact mechanism is not clear (Sha et al., 2021). The relationship between ferroptosis and DFU has rarely been reported, but the correlation does exist. Inhibitors related to ferroptosis had been shown to play a protective role in the diabetic foot (He et al., 2022). In addition, studies had shown that paeoniflorin (PF) has the ability to promote DFU wound healing by activating the *NRF2* related ferroptosis pathway and the *NRF2/HO-1* pathway (Sun et al., 2020). Therefore, exploring the pathogenesis of DFU due to ferroptosis may provide new therapeutic targets for the treatment of DFU and other chronic wounds. Our study obtained 25 ferroptosis genes associated with DFU through the WGCNA and FerrDb databases.

Machine learning has a wide range of applications in the biomedical field, demonstrating excellent efficiency in clinical diagnosis and optimal treatment (Huang et al., 2022). Subsequently, we applied machine learning algorithms to screen key ferroptosis genes from ferroptosis-related genes associated with DFU, and finally identified two ferroptosis-related genes (*MAFG* and *MAPK3*) as potentially effective diagnostic molecules for DFU.

It has been clearly reported that MAF BZIP Transcription Factor G *(MAFG)* has a promising potential as a potential prognostic biomarker in non-small cell lung cancer. *MAFG* also can play a role as a molecular biomarker for tumor-targeted therapy to relieve cisplatin resistance of tumor cancer cells, improving therapeutic and prognostic efficiency (Vera-Puente et al., 2018). However, there are few studies related to *MAFG* in the pathogenesis of DFU progression, and only a few reports suggest that *MAFG* loss improves glucose metabolism and insulin sensitivity, thus protecting from hyperglycemia (Pradas-Juni et al., 2020). Our results demonstrated that *MAFG* was highly expressed in DFU samples compared to controls, and we could hypothesize that the high expression of *MAFG* had a detrimental effect in DFU. Nevertheless, the specific mechanism of *MAFG* in DFU is unclear and further relevant studies are required to determine whether it can be used as the therapeutic target.

External dataset validation was employed to assess the efficiency of both genes as therapeutic target, suggesting that the accuracy and specificity of *MAPK3* was superior to that of *MAFG.* (Mitogen-Activated Protein Kinase 3) *MAPK3*, also known as extracellular signal-regulated kinase 1 (*ERK1*), is a protein-coding gene that encodes a protein belonging to the *MAP* kinase family (www.ncbi.nlm.nih.gov/gene/), which are involved in a variety of cellular processes such as proliferation, inflammation, and cellular metabolism through phosphorylation of their target proteins (Kassouf and Sumara, 2020). In this study, high expression of *MAPK3* played a positive role in variety of the pathways associated with cell proliferation and differentiation, such as *MAPK* signaling pathway, growth hormone synthesis, secretion and action, *ErbB* signaling pathway, and *GnRH* signaling pathway. However, some studies have shown that overactivation of *ERK1/2* is associated with deleterious effects during obesity and diabetes. The absence of *ERK1/2* in the liver improves systemic insulin and glucose tolerance (Jiao et al., 2013). It has been recently shown that high glucose-activated *ERK1/2* increases matrix metalloproteinase 9 (*MMP9*) expression in the skin and contributes to the delayed healing of DFU wounds (Lang et al., 2021). Additionally, reactive oxygen species (ROS)-triggered activation of *ERK1/2* is involved in the NETosis process, and diabetes-induced neutrophil NETosis disrupts wound healing through neutrophil extracellular traps (NETs) (Yang et al., 2019). Accordingly, we deduced that high *MAPK3* expression has a delayed wound healing effect on DFU patients. Considering the adverse effects of *MAPK3*, corresponding new molecular therapeutic strategies should also be developed.

However, there are limitations to this study. Firstly, the machine learning prediction model in the external validation cohort affects the accuracy due to the small sample size, leading to misdiagnosis and missed diagnoses. Thus, the larger DFU sample size can improve the prediction accuracy. Secondly, the ferroptosis-related biomarkers identified in this study that have the potential to be therapeutic targets for DFU require further literature support and basic experimental validation. FerrDb database is constantly being updated, and more ferroptosis-related genes are yet to be discovered.

## Data Availability

The original contributions presented in the study are included in the article/Supplementary Material, further inquiries can be directed to the corresponding authors.
