# Supplementary material for: Revealing the novel ferroptosis-related therapeutic targets for diabetic foot ulcer based on the machine learning
Source: Front Genet. 2022 Sep 26;13:944425. doi: 10.3389/fgene.2022.944425 (PMC9549267; doi:10.3389/fgene.2022.944425)
Supplement: Supplementary file 1 [file DataSheet1.zip › Supplementary Material/Supplementary Material.docx]

Supplementary Material

# Supplementary Tables

Supplementary Table 1: The results of GO analysis of 146 DFU-related genes.

Supplementary Table 2: The results of KEGG analysis of 146 DFU-related genes.

Supplementary Table 3: The results of GO, DO, and KEGG analysis of hub genes.

# Supplementary Figures


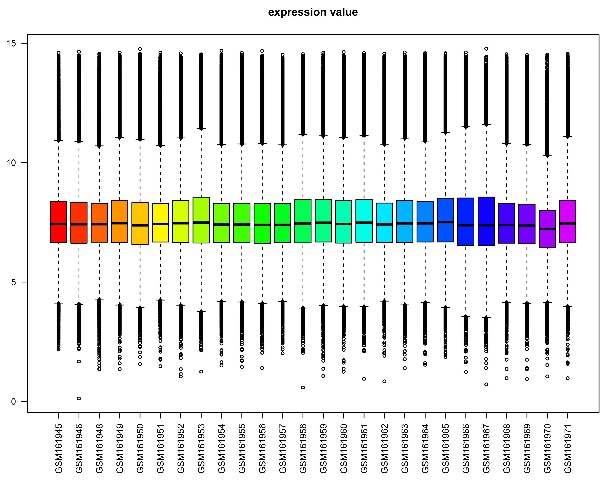

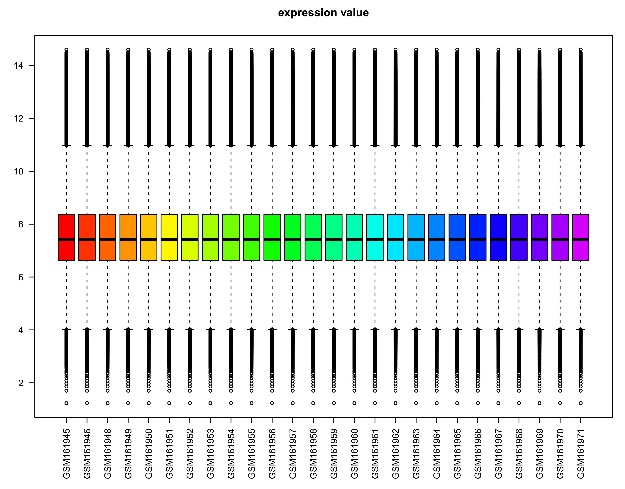


**(A) (B)**


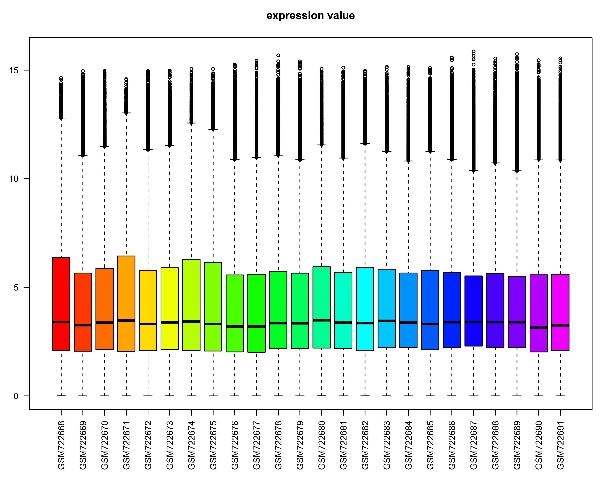

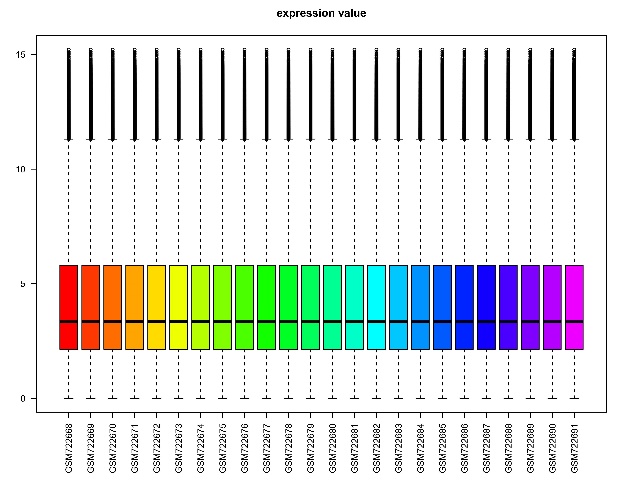


**(C) (D)**

Supplementary Figure 1: Normalization of gene expression data in samples

(A)Before normalization of GSE7014. (B) After normalization of GSE7014.

(C) Before normalization of GSE29221. (D) After normalization of GSE29221.
