# Supplementary figures and images for: Revealing the novel ferroptosis-related therapeutic targets for diabetic foot ulcer based on the machine learning
Source: Front Genet. 2022 Sep 26;13:944425. doi: 10.3389/fgene.2022.944425 (PMC9549267; doi:10.3389/fgene.2022.944425)

expression value

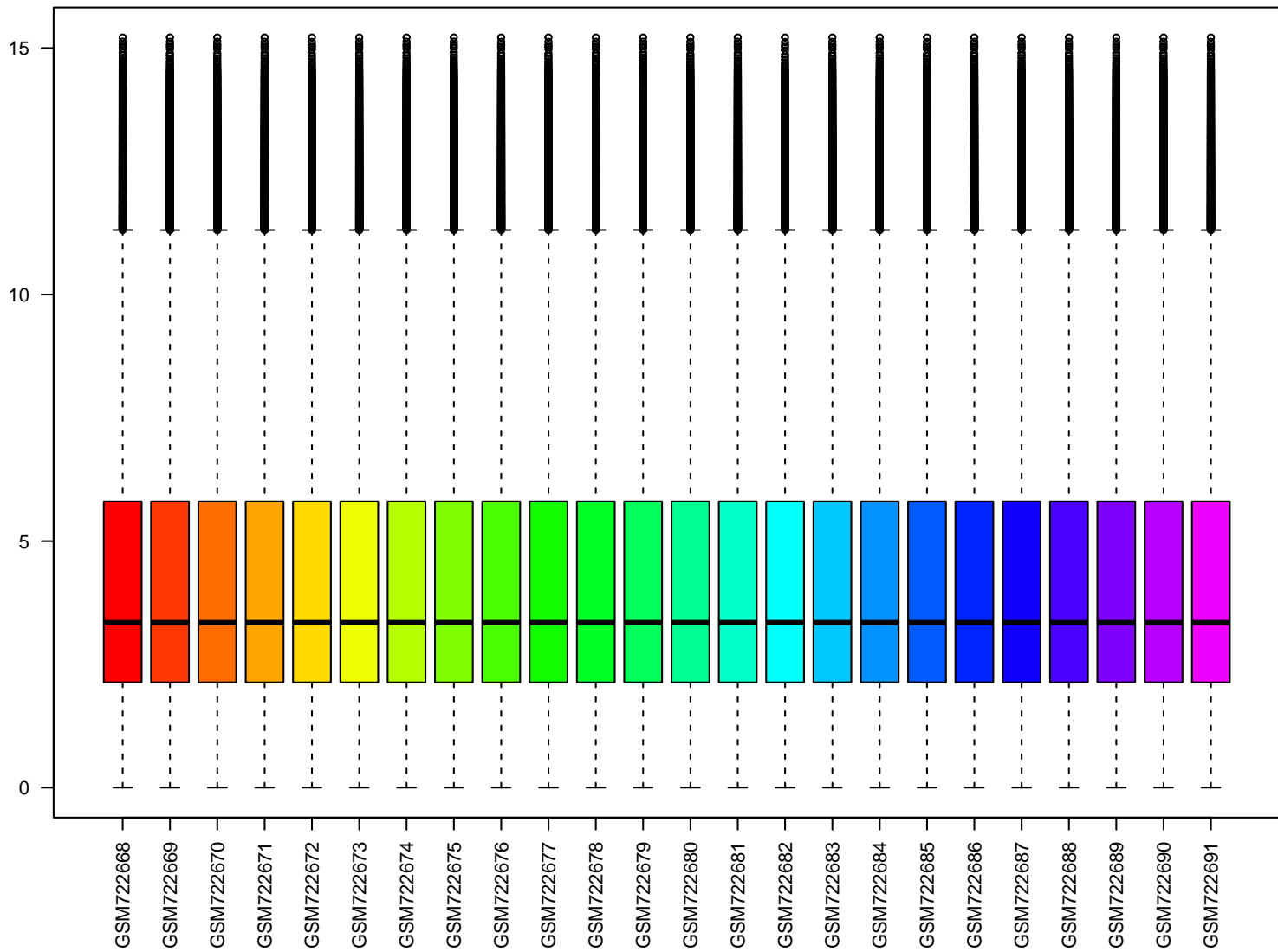

Supplement: Supplementary file 1 [file DataSheet1.zip › Supplementary Material/Supplementary Figure 1/GSE29221-after-normalization.pdf]

expression value

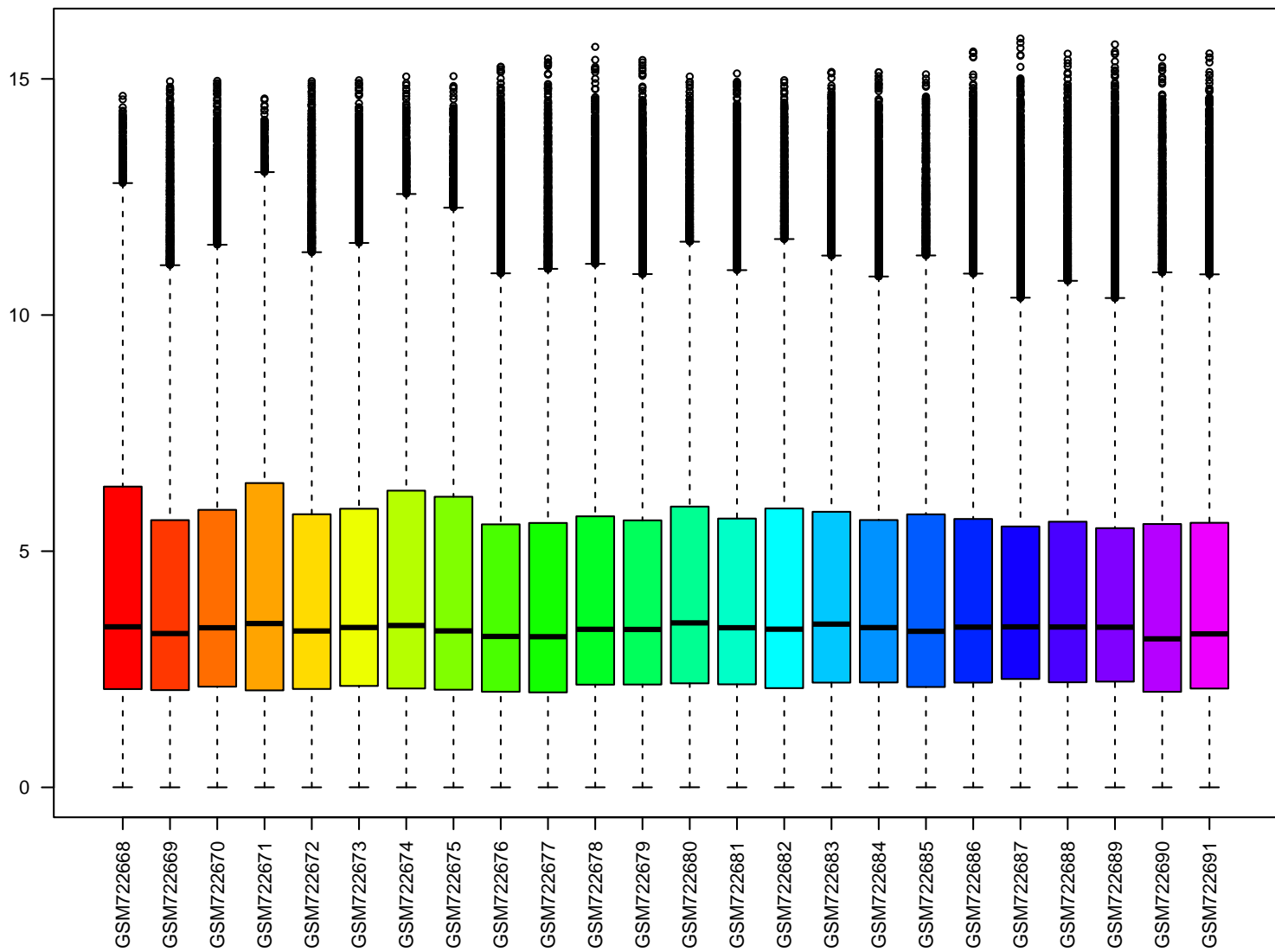

Supplement: Supplementary file 1 [file DataSheet1.zip › Supplementary Material/Supplementary Figure 1/GSE29221-pre-normalization.pdf]

expression value

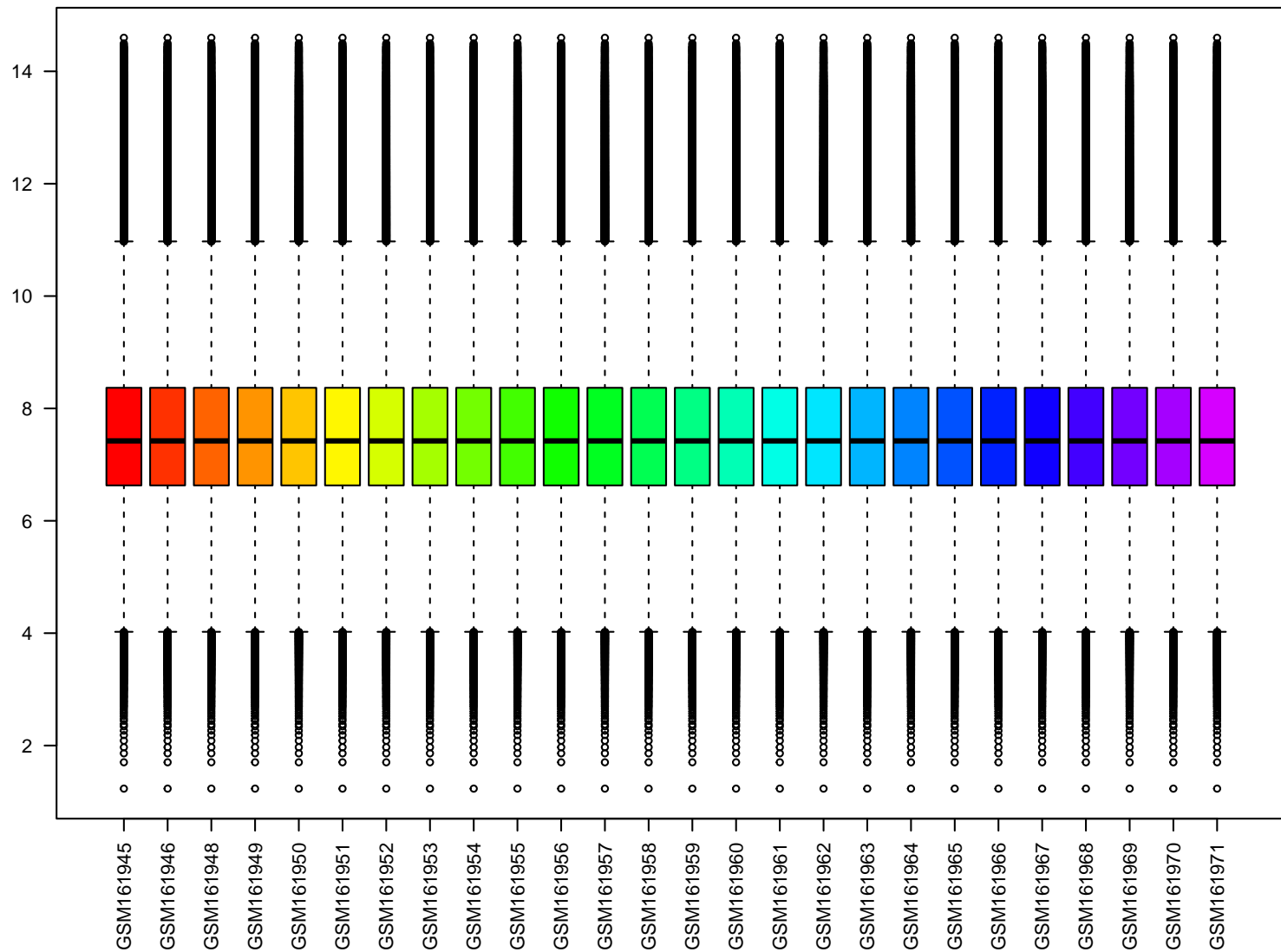

Supplement: Supplementary file 1 [file DataSheet1.zip › Supplementary Material/Supplementary Figure 1/GSE7014-after-normalization.pdf]

expression value

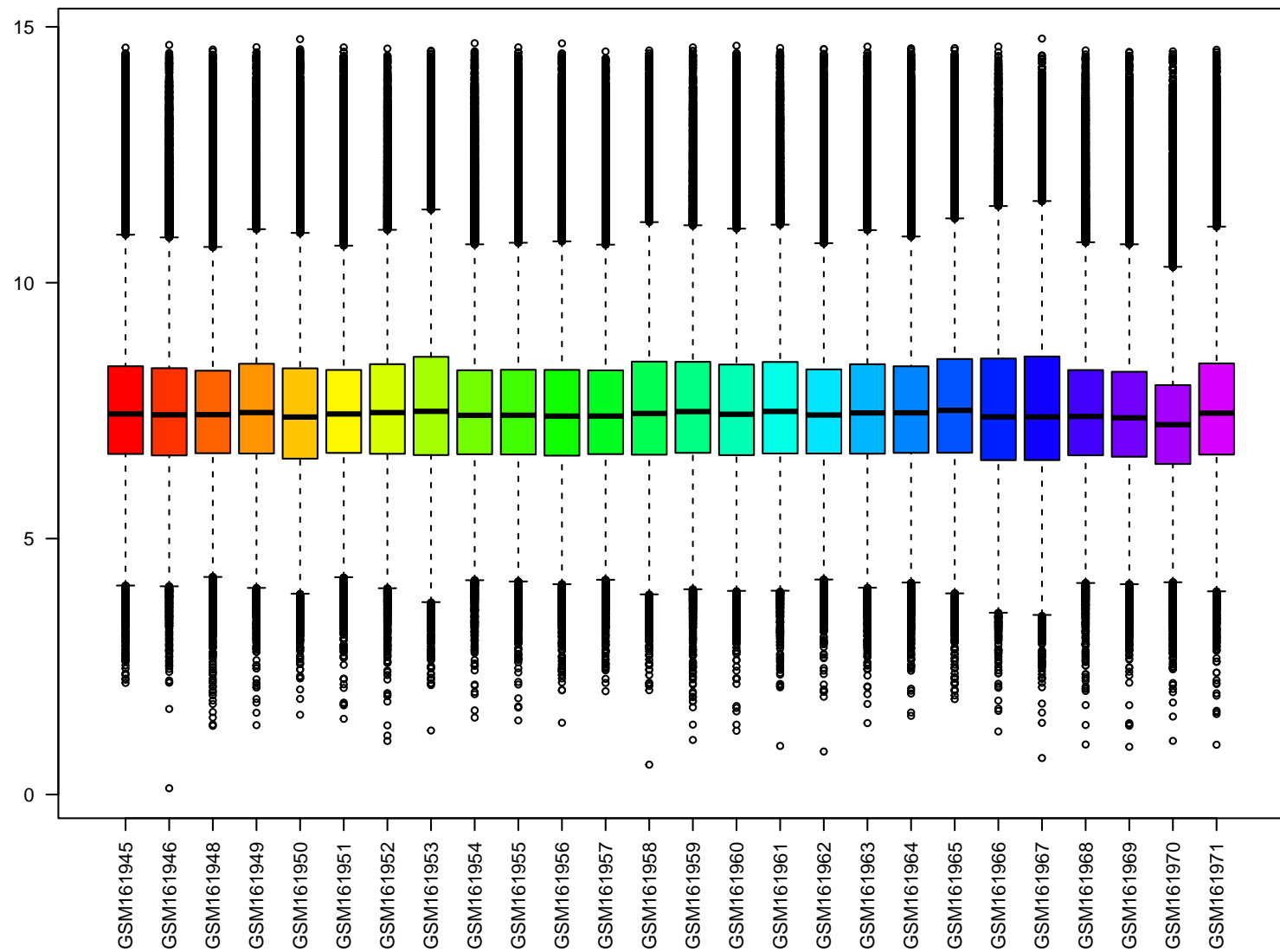

Supplement: Supplementary file 1 [file DataSheet1.zip › Supplementary Material/Supplementary Figure 1/GSE7014-pre-normalization.pdf]
